# Distinct Molecular Landscape and Clinical Subtype of HPV-Positive Esophageal Squamous Cell Carcinoma

**DOI:** 10.3390/cancers18193103

**Published:** 2026-09-24

**Authors:** Hang Li, Xuxia Shen, Yan Jin, Hong Hu, Jiaqing Xiang, Yawei Zhang

**Affiliations:** 1 Departments of Thoracic Surgery and State Key Laboratory of Genetic Engineering, Fudan University Shanghai Cancer Center, Shanghai 200032, China; 2 Institute of Thoracic Oncology, Fudan University, Shanghai 200032, China; 3 Department of Oncology, Shanghai Medical College, Fudan University, Shanghai 200032, China; 4 Department of Pathology, Fudan University Shanghai Cancer Center, Shanghai 200032, China

**Keywords:** esophageal squamous cell carcinoma, human papillomavirus, PTEN, PIK3CA, molecular subtype, prognosis

## Abstract

**Background**: This study aimed to investigate the prevalence of human papillomavirus (HPV) infection in Chinese patients with esophageal squamous cell carcinoma (ESCC) and its association with driver gene mutations (EGFR, KRAS, PIK3CA, PTEN), clinicopathological features, and prognosis. **Methods**: A retrospective cohort of 120 Chinese ESCC patients was analyzed. HPV infection status and typing were determined by polymerase chain reaction (PCR)-based reverse hybridization in tumor tissues; EGFR, KRAS, PIK3CA, and PTEN mutations were assessed by Sanger sequencing. Associations with clinicopathological variables and survival were evaluated. **Results**: The HPV infection rate was 44.2%, with 37.5% single-type and 6.7% mixed infections, predominantly high-risk types. No EGFR or KRAS mutations were detected, whereas PTEN and PIK3CA mutations were found in 16.7% and 6.7% of patients, respectively, and were mutually exclusive. HPV-negative tumors more frequently showed lymphovascular invasion (*p* = 0.03) and PTEN mutations (*p* = 0.017). HPV positivity was associated with better recurrence-free survival and overall survival. **Conclusions**: HPV infection is prevalent in Chinese ESCC and may delineate a distinct molecular profile; HPV-negative ESCC was more frequently associated with aggressive clinicopathological features, including lymphovascular invasion and PTEN mutations. These findings support subtype-stratified prevention and treatment strategies for ESCC.

## Simple Summary

Human papillomavirus (HPV) is a well-established cause of several anogenital and oropharyngeal cancers, but its role in esophageal squamous cell carcinoma (ESCC) among Chinese patients remains controversial. We retrospectively analyzed 120 Chinese patients with ESCC and detected HPV DNA in 44.2% of tumors (53/120), predominantly high-risk types such as HPV16 and HPV18. HPV-negative tumors more frequently carried PTEN mutations and exhibited lymphovascular invasion, whereas HPV positivity was associated with a trend toward better recurrence-free survival. These findings suggest that HPV DNA status may delineate subgroups of ESCC with distinct clinicopathological and molecular features and could inform subtype-stratified prevention and treatment strategies; however, the transcriptional activity of the detected HPV and its causal contribution to ESCC require further investigation.

## 1. Introduction

Esophageal cancer presents a significant global health challenge, ranking as a major malignancy with substantial incidence and mortality rates [1,2]. China, which bears a disproportionately large burden of the disease, has historically accounted for approximately half of the world’s esophageal cancer cases [3,4]. These statistics indicate that addressing risk factors and improving healthcare accessibility are key to reducing the burden of esophageal cancer in China and many other countries in the world.

Esophageal cancer presents a striking global disparity in its histopathological presentation. In China, the vast majority of patients are diagnosed with esophageal squamous cell carcinoma (ESCC), while in Western countries such as the United States and those in Europe, esophageal adenocarcinoma predominates [5,6]. The risk factors underlying this disparity in pathological types have consistently sparked considerable discussion.

Human papillomavirus (HPV) infection is strongly associated with the development of squamous cell carcinomas in various organs. High-risk HPV types, such as HPV16 and HPV18, are causative agents in the majority of cervical cancers [7,8,9]. Furthermore, HPV is implicated in a significant proportion of head and neck squamous cell carcinomas, particularly those arising in the oropharynx [10,11]. Additionally, HPV infection is a well-established risk factor for anal squamous cell carcinoma [9]. The oncogenic mechanisms of HPV involve the E6 and E7 oncoproteins, which interfere with cellular tumor suppressor proteins, leading to uncontrolled cell proliferation and genomic instability [12,13]. Understanding the specific HPV types and their prevalence in different squamous cell carcinomas is crucial for developing targeted prevention and treatment strategies. Reported HPV prevalence in ESCC varies widely, and this heterogeneity is largely explained by geographic origin, with higher rates reported in Asia, including China, than in Europe, North America and Oceania [14]. Pooled case–control data indicate an approximately threefold increase in the odds of HPV DNA detection in ESCC tissue relative to controls [15]; nevertheless, because most constituent studies assessed HPV DNA alone, the available evidence is still considered insufficient to establish HPV as a prominent carcinogen in ESCC [14].

In this study, we focus on the frequency of HPV infection and well-known driver molecular alterations, including EGFR, KRAS, PIK3CA and PTEN gene mutations, in Chinese esophageal squamous cell cancer patients. We also reveal the association between HPV infection and clinical characteristics, as well as prognosis. Our findings characterize the molecular and clinicopathological features associated with HPV DNA positivity in Chinese ESCC and may inform future cancer-prevention programs, including vaccination or screening strategies.

## 2. Materials and Methods

### 2.1. Patients and Specimen Collection

Patients were enrolled retrospectively if they had newly diagnosed primary esophageal squamous cell carcinoma (ESCC) between April 2019 and April 2020. All patients provided written informed consent for the surgical procedure and underwent radical surgery. The eligibility criteria were as follows: (i) the diagnosis of ESCC was confirmed by central pathological re-review carried out independently by two pathologists (X.S. and Y.J.); (ii) sufficient tumor tissue together with the corresponding histologically normal mucosa was available for analysis; (iii) no neoadjuvant treatment was given before surgery; and (iv) an R0 resection was achieved in every case. The study was approved by the ethics committee of Fudan University Shanghai Cancer Center (approval No. 2023-126-1218). Because this was a retrospective study using de-identified archived specimens, the requirement for informed consent for research use of these specimens was waived.

### 2.2. HPV Detection

Following surgery, each specimen was divided into two portions: one portion was fixed in formalin and embedded in paraffin for histopathological sectioning, and the other was snap-frozen in liquid nitrogen immediately after resection and stored in liquid nitrogen. Total RNA and DNA were co-extracted from the frozen tumor tissue and from distant, histologically normal esophageal mucosa with a DNA/RNA co-extraction kit (Tiangen, Beijing, China), after the frozen specimens had been disrupted in TRIzol reagent (Life Technologies, Carlsbad, CA, USA).

Extracted DNA was added in a mixed PCR system (Yaneng Bio, Shenzhen, China), which contained the 5′ biotinylated MY09-MY11-HMB01 L1 consensus primer system (type 6, 11, 16, 18, 31, 33, 35, 39, 42, 43, 45, 51, 52, 56, 58, 59, 66, 68, 73, 82, 83), 5′ biotinylated GH20/PC04 human β-globin primer, and Taq DNA polymerase. Human β-globin DNA was applied to quality control. The PCRs were amplified for 40 cycles in the Eppendorf Vapo protect system (Eppendorf, Hamburg, Germany).

HPV detection and typing analysis were performed by PCR dot-blot reverse hybridization [16,17]. Oligonucleotide probes were immobilized on a nylon membrane strip (Yaneng Bio, Shenzhen, China), which was hybridized with 25 μL PCR product in 5 mL hybridization buffer at 53 °C for 90 min. After hybridization, every membrane was washed 3 times in 20 mL washing buffer at 53 °C to remove nonspecifically bound DNA and optimize specific hybridization. Following stringent washing, 3 mL of streptavidin–horseradish peroxidase solution was applied to cover each membrane in a tray, which was placed on a 60 rpm rotating platform at room temperature for 30 min. Unbound peroxidase solution was removed by two 10 min washes in 20 mL washing buffer, then the membrane was rinsed in 0.1 M sodium citrate. Color development was activated by incubation in a 10:1 mixture of substrates A (hydrogen peroxide in sodium citrate buffer) and B (3,3′,5,5′-tetramethylbenzidine in dimethylformamide) for 30 min in a dark environment. After color development, the membrane was rinsed in deionized water and dried for photographing and analysis (Figure 1).

### 2.3. Mutation Analysis

*EGFR* (exons 18 to 21), *KRAS* (exons 2 to 3), *PIK3CA* (exon 9 and exon 20) and *PTEN* (whole exon) were amplified by polymerase chain reaction (PCR) using cDNA; the principle of the PCR method was described elsewhere [18].

cDNA-derived PCR products were analyzed by direct Sanger sequencing in both the forward and reverse directions [19]. All identified mutations were confirmed by sequencing an independent PCR product; when multiple mutations coexisted in the cDNA-derived product, individual exons were re-amplified and genotyped separately using genomic DNA.

### 2.4. Clinical and Pathological Variables

The clinical and pathological variables retrieved for this analysis comprised sex, age at diagnosis, smoking history, lesion location, lymphovascular space invasion, lymph node metastasis, and pathological TNM stage. Lymphovascular space invasion was assessed by histopathological examination of tumor sections, defined as the presence of free tumor cell clusters within the lumen of vascular or lymphatic channels. Preoperative staging relied on contrast-enhanced computed tomography (CT) of the chest together with magnetic resonance imaging (MRI) of the brain and abdomen; the same examinations were repeated after surgery to detect local recurrence or distant metastasis. Pathological TNM stage was assigned according to the eighth edition of the esophageal cancer staging classification system. After the date of diagnosis, patients were interviewed every three months, either in the outpatient clinic or by telephone, regarding disease recurrence and survival. Recurrence-free survival (RFS) was defined as the interval from diagnosis to recurrence or the last follow-up, and overall survival (OS) as the interval from diagnosis to death or the last follow-up. The median follow-up of the cohort was 45.2 months.

### 2.5. Statistical Analysis

Qualitative variables are reported as percentages, and quantitative variables as means with standard deviations. Categorical associations were compared with Pearson’s chi-square test or Fisher’s exact test, as appropriate. Recurrence-free and overall survival were estimated with the Kaplan–Meier method and compared by the log-rank test. Multivariate survival analysis was performed using Cox proportional hazards regression models, including age at diagnosis, smoking history, tumor location, PTEN mutation, PIK3CA mutation, and HPV infection status; results are reported as hazard ratios (HRs) with 95% confidence intervals. All analyses were carried out with SPSS for Windows (version 19.0). All tests were two-tailed, and statistical significance was defined as *p* < 0.05.

## 3. Results

A total of 120 patients with primary ESCC, whose age at diagnosis ranged from 37 to 76 years old, were consecutively collected from April 2019 to April 2020 for our study. HPV status was determined in 44.2% of these patients (53 out of 120). In total, 45 (37.5%) patients had single-type HPV infection and 8 (6.7%) patients had mixed HPV involvement (Table 1). According to previous studies about HPV-mediated carcinogenesis [20,21], all the HPV types detected in our series were high-risk types. The detailed distribution of HPV genotypes, including single and mixed infections, is presented in Supplementary Table S1.

Neither EGFR nor KRAS gene mutation was found, while 20 (16.67%) PTEN mutations and 8 (6.67%) PIK3CA mutations were detected from 120 patients. PTEN mutation and PIK3CA mutation were mutually exclusive. E545K (n = 3) was the most frequent subtype of PIK3CA mutation, followed by E542K (n = 2), H1047R (n = 2) and F980I (n = 1). Multiple-point mutations in the PTEN gene were a common phenomenon and the most common subtype of PTEN mutation was I224M, which was harbored in 95% patients with PTEN mutation.

Furthermore, the association between clinical characteristics, molecular alterations and HPV-infected status was investigated. No association was found with sex, age at diagnosis, smoking history, lesion location, lymph node metastasis, and pTNM stage. Lymphvascular invasion and PTEN mutation occurred significant more frequently in ESCC patients without HPV infection (*p* = 0.03, *p* = 0.017) (Table 2).

The effect of HPV-infected status, as well as PTEN and PIK3CA mutation, on survival in ESCC patients was further evaluated. Better 5-year RFS (35.6% vs. 22.8%, log-rank *p* = 0.039) and OS (47.2% vs. 35.5%, log-rank *p* = 0.042) were found in patients with HPV infection (Figure 2). The influence of gene mutation on clinical outcome was observed in patients with PTEN or PIK3CA mutation; however, it did not reach statistical significance. Cox Regression showed age at diagnosis, smoking history, tumor location, PIK3CA mutation and PTEN mutation had no significant influence on the OS or RFS of these patients. Meanwhile, HPV-infected status showed its significance in predicting better RFS (HR, 0.373; 95%CI, 0.201–0.682, *p* = 0.033) and OS (HR, 0.421; 95%CI, 0.237–0.911, *p* = 0.041) in multivariate analysis.

## 4. Discussion

Esophageal squamous cell carcinoma remains a significant global health challenge, particularly in China, which accounts for approximately half of the world’s esophageal cancer cases. This study aimed to investigate the prevalence of HPV infection and common driver molecular alterations, including EGFR, KRAS, PIK3CA, and PTEN gene mutations, in a Chinese cohort of ESCC patients, and to explore their associations with clinicopathological characteristics and prognosis.

Our findings reveal a high prevalence of HPV infection in ESCC patients from this Chinese cohort, with 44.2% of patients testing positive for HPV, primarily high-risk types. This aligns with previous reports from endemic regions like China and Middle East countries [22,23]. The observed HPV prevalence is notably higher than the typically reported rates of less than 10–15% in Western ESCC cohorts [23,24], reinforcing the hypothesis that HPV may play a more significant role in ESCC pathogenesis in geographically distinct populations with different environmental exposures and genetic backgrounds [25,26]. Data from other HPV-associated head and neck sites in China provide useful context. A meta-analysis of 2896 Chinese patients with head and neck cancer estimated an HPV16 prevalence of 24.7%, rising to 31.6% in oropharyngeal cancer [27], and a recent series of 388 oropharyngeal squamous cell carcinomas from eastern China reported p16 positivity in 62.6% of cases, increasing from 45.5% in 2017 to 69.5% in 2023 [28]. HPV prevalence therefore differs substantially among anatomical sites and is changing over time within the same population, a context that should be considered when interpreting the 44.2% HPV DNA positivity observed in the present ESCC cohort. By contrast, the prognostic significance of HPV in non-oropharyngeal sites remains uncertain, and in the oral cavity, squamous cell carcinoma HPV DNA is detected in only approximately 2% of tumors and does not affect outcome [29,30]. The detection of both single-type (37.5%) and mixed HPV infections (6.7%) further underscores the complexity of HPV’s involvement in ESCC. High-risk HPV types, particularly HPV16 and HPV18, are known causative agents in various squamous cell carcinomas, including cervical and oropharyngeal cancers, through their E6 and E7 oncoproteins which dysregulate tumor suppressor pathways [31,32,33]. The specific mechanisms involve E6 promoting p53 degradation and E7 inactivating the retinoblastoma protein (pRb), leading to uncontrolled cell proliferation and genomic instability [33,34,35,36].

In terms of molecular alterations, our study found no EGFR or KRAS gene mutations in the ESCC cohort, which is consistent with the established literature indicating these alterations are rare in ESCC, unlike in other cancers such as lung or colorectal adenocarcinomas [37,38]. This highlights the distinct oncogenic drivers present in squamous epithelia. EGFR, KRAS, PIK3CA and PTEN were selected for analysis because they belong to the RTK–RAS and PI3K–AKT–mTOR pathways, which are potentially targetable, and because PTEN and PIK3CA have both been reported as recurrently mutated genes in ESCC [39]. By contrast, the genes most frequently mutated in large-scale ESCC sequencing studies, such as TP53, NOTCH1, NFE2L2 and CDKN2A, are predominantly tumor suppressors or squamous-lineage genes without current targeted therapies [40]. In contrast, PTEN mutations were detected in 16.7% of patients and PIK3CA mutations in 6.7% of patients. Notably, PTEN and PIK3CA mutations were mutually exclusive, suggesting a convergent dysregulation of the PI3K-AKT-mTOR signaling pathway, which is a hallmark of ESCC progression [41]. The most frequent PIK3CA mutation subtype was E545K (n = 3), followed by E542K (n = 2), H1047R (n = 2), and F980I (n = 1). The PTEN mutations frequently involved multiple-point mutations, with I224M being the most common subtype, found in 95% of patients with PTEN mutations. PIK3CA and PTEN alterations are also recurrent in other HPV-associated squamous cancers, most notably cervical carcinoma [42], and aberrations of PIK3CA are enriched in HPV-positive head and neck squamous cell carcinoma [43], suggesting that dysregulation of the PI3K–AKT–mTOR pathway is a shared feature across these tumor types rather than a finding unique to ESCC.

A significant finding was the association between HPV negativity and certain aggressive clinicopathological features. Lymphovascular invasion (LVI) and PTEN mutation occurred significantly more frequently in ESCC patients without HPV infection (*p* = 0.03 and *p* = 0.017, respectively). The presence of LVI is a critical prognostic factor, indicating a higher risk of metastasis. This suggests that HPV-negative ESCC may follow a more aggressive clinical course, consistent with the poorer outcomes reported in some earlier studies [34,37]. Other clinical characteristics, including sex, age at diagnosis, smoking history, lesion location, and pTNM stage, did not show significant associations with HPV infection status or specific gene mutations.

Although our study did find a statistically significant association between HPV status and survival, this observation warrants further validation in larger, prospective, multicenter cohorts with extended follow-up periods. Previous studies have shown conflicting results regarding the prognostic role of HPV in ESCC, with some indicating a better prognosis for HPV-positive cases [44,45], while others find no significant impact [46]. These discrepancies may arise from variations in geographical populations, HPV detection methodologies, and patient cohorts.

Collectively, these findings suggest that HPV infection may delineate a biologically distinct subgroup of ESCC, although functional evidence is needed to confirm its biological relevance. The higher prevalence of HPV in Chinese ESCC patients suggests a potential for HPV-targeted prevention strategies, such as vaccination programs, in high-incidence regions [22]. Furthermore, the immunogenic nature of viral antigens in HPV-driven cancers could make these tumors more amenable to immune checkpoint blockade therapies [34,47]. The mutually exclusive nature of PTEN and PIK3CA mutations points to convergent dysregulation of the PI3K-AKT-mTOR pathway in a subset of ESCC; notably, only PTEN mutations, not PIK3CA mutations, were enriched in HPV-negative tumors in this cohort. This pathway may nonetheless represent a relevant therapeutic target, particularly for HPV-negative cases, which appear to follow a more aggressive course.

A limitation of this study is its retrospective design and the sample size from a single institution, which may limit the generalizability of some findings, especially regarding survival associations. In addition, direct Sanger sequencing has limited sensitivity for low-frequency variants, with a practical detection limit of approximately 15–20% variant allele frequency; the mutation frequencies reported in this study may therefore represent underestimates. Future studies should integrate whole-exome sequencing, RNA expression profiling, and comprehensive analyses of the tumor immune microenvironment to further elucidate the intricate interplay between HPV infection, PTEN/PIK3CA alterations, and antitumor immunity in ESCC. Such comprehensive analyses will be crucial for developing more precise diagnostic, prognostic, and therapeutic strategies tailored to the distinct molecular subtypes of ESCC.

In addition, HPV status in this study was defined by DNA detection alone. Because residual tumor RNA was not available, we could not evaluate the transcriptional activity of HPV (e.g., E6/E7 mRNA expression), surrogate markers such as p16INK4a immunohistochemistry, viral load, or viral integration status. HPV DNA positivity therefore cannot be equated with transcriptionally active, causal infection, and our findings should be interpreted as associations between HPV DNA status and the clinical and molecular features examined.

## 5. Conclusions

This study characterized the prevalence of HPV infection and the profile of selected driver gene alterations (PTEN and PIK3CA) in a cohort of Chinese patients with ESCC. HPV DNA was detected at a relatively high frequency in this cohort. Notably, HPV-negative tumors were more frequently associated with aggressive clinicopathological features, including lymphovascular invasion and PTEN mutations. HPV positivity was associated with better RFS and OS, although the survival analyses were limited by the sample size and the duration of follow-up. These findings suggest that HPV DNA status may define clinically relevant subgroups of ESCC, with potential implications for stratified prevention and therapeutic strategies; nonetheless, the transcriptional activity of the detected HPV and its causal role in ESCC remain to be established. Future research integrating multi-omics approaches is warranted to further characterize the interplay between HPV infection, genetic alterations, and the tumor microenvironment, and to advance precision diagnostics and personalized treatment for ESCC.

## Figures and Tables

**Figure 1:**
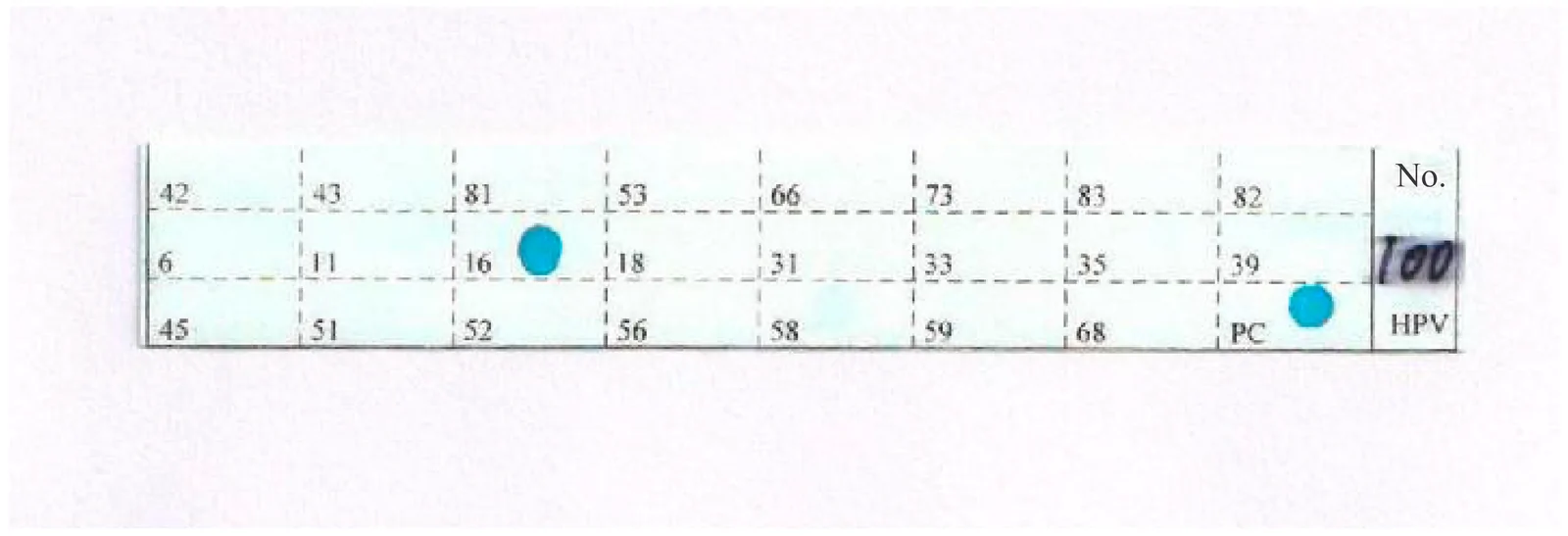
Representative HPV genotyping results by PCR dot-blot reverse hybridization. Patient IDs are shown at the top; rows indicate HPV type-specific probes; blue dots denote positive hybridization signals. PC, positive control.

**Figure 2:**
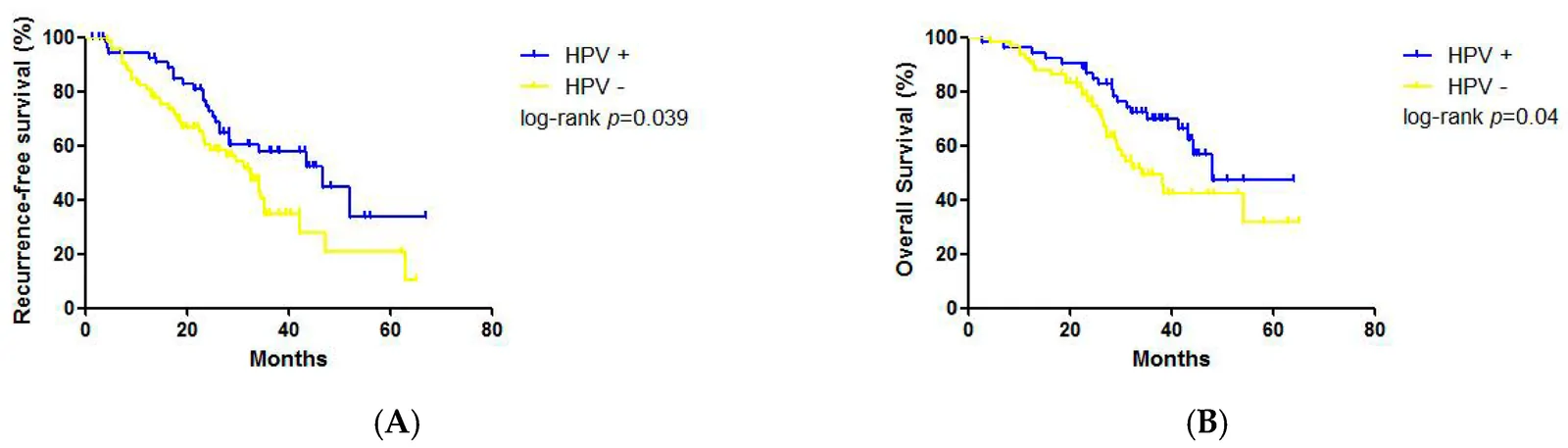
Kaplan–Meier survival analysis of ESCC patients stratified by HPV infection status. (**A**) Recurrence-free survival (RFS) and (**B**) overall survival (OS).

**Table 1:** The clinical characteristics and HPV infection status of patients in the cohort.

Variables	No.	%
Sex		
Male	111	92.5
Female	9	7.5
Age		
≥60 years old	66	55.0
<59 years old	54	45.0
Smoking History		
Smoker	74	61.7
Never smoker	46	38.3
Smoking Amount		
≤20 pack-years	26	35.1
>20 pack-years	48	64.9
Lesion Location		
Upper and middle	74	61.7
Lower	46	38.3
TNM Stage		
I/II	48	40.0
III	72	60.0
N Stage		
N0	46	38.3
N1–3	74	61.7
Lymphvascular invasion		
Positive	37	30.8
Negative	83	69.2
HPV infection		
Positive	53	44.2
Negative	67	55.9
Infection Status		
Single	45	84.9
Mixed	8	15.1

Abbreviations: HPV, human papillomavirus.

**Table 2:** Comparison of clinical characteristics between HPV-positive and HPV-negative esophageal cancer patients.

	HPV-Positive No. (%)	HPV-Negative No. (%)	*p*
Sex			
Male	49 (92.4)	62 (92.5)	1
Female	4 (7.6)	5 (7.4)	
Age (years old)			
Mean ± SD	60.1 ± 0.9	59.3 ± 1.1	0.870
Smoking History			
Smoker	29 (54.7)	45 (67.2)	0.164
Never	24 (45.3)	22 (32.8)	
Lesion Location			
Upper and middle	35 (66.0)	39 (58.2)	0.381
Lower	18 (34.0)	28 (41.8)	
TNM Stage			
I/II	24 (45.3)	24 (35.8)	0.293
III	29 (54.7)	43 (64.2)	
N stage			
N0	21 (39.6)	25 (37.3)	0.796
N1–3	32 (60.4)	42 (62.7)	
PIK3CA			
Mutation	3 (5.7)	5 (7.5)	1.000
Wild-type	50 (94.3)	72 (92.5)	
PTEN			
Mutation	4 (7.5)	16 (23.9)	0.017
Wild-type	49 (92.5)	51 (76.1)	
Lymphvascular invasion			
Positive	11 (20.7)	26 (38.8)	0.030
Negative	42 (79.3)	41 (61.2)	

Abbreviations: HPV, human papillomavirus; PIK3CA, phosphatidylinositol-4,5-bisphosphate 3-kinase catalytic subunit alpha; PTEN, phosphatase and tensin homolog deleted on chromosome ten.

## Data Availability

The data presented in this study are available on request from the corresponding author. The data are not publicly available due to privacy and ethical restrictions.

## Supplementary Materials

The following supporting information can be downloaded at https://www.mdpi.com/article/10.3390/cancers18193103/s1, Supplementary Table S1: Distribution of HPV genotypes among the 53 HPV DNA-positive ESCC patients.
