# Supplementary material for: Distinct Molecular Landscape and Clinical Subtype of HPV-Positive Esophageal Squamous Cell Carcinoma
Source: Cancers (Basel). 2026 Sep 24;18(19):3103. doi: 10.3390/cancers18193103 (PMC13664334; doi:10.3390/cancers18193103)
Supplement: Supplementary file 1 [file cancers-18-03103-s001.zip › cancers-4548627-supplementary.pdf]

Supplementary Table S1. Distribution of HPV genotypes among the 53 HPV DNA-positive ESCC patients.

HPV genotype	Single infection, n	Mixed infection, n	Total, n	% of HPV-positive patients (n = 53)
HPV 16	41	8	49	92.5
HPV 18	4	4	8	15.1
HPV 58	0	3	3	5.7
HPV 59	0	1	1	1.9
HPV 66	0	1	1	1.9
Any genotype	45	8	53	100.0

Infection status: single infection, n = 45 (84.9%); multiple infections, n = 8 (15.1%). The mixed infections comprised HPV 16 + 18 (n = 3), HPV 16 + 58 (n = 2), HPV 16 + 59 (n = 1), HPV 16 + 18 + 58 (n = 1), and HPV 16 + 66 (n = 1). HPV, human papillomavirus; ESCC, esophageal squamous cell carcinoma.
